# Laparoendoscopic Single-Site Surgery (LESS) for a Large Ovarian Tumour: First Clinical Case Report

**DOI:** 10.1155/2011/105643

**Published:** 2011-02-15

**Authors:** Yao Dong Chua, Ying Woo Ng, Yoke Fai Fong

**Affiliations:** Department of Obstetrics and Gynaecology Division of Benign Gynaecology, National University Hospital, Nuhs Tower Block Level 12, 1E kent Ridge Road, Singapore S(119228)

## Abstract

*Objective*. To report the feasibility of removing a 10 cm ovarian fibroma via a laparoendoscopic single-site trocar through trans-umbilical access. *Design*. Case report. *Setting*. Teaching and research hospital. *Patient*. A 64-year-old patient affected by a large 10 cm ovarian tumour. *Intervention(s)*. Bilateral salpingo-oophorectomy a large 10 cm ovarian tumour, using a laparoendoscopic single-site approach with a Covidien SILS multitrocar access device and standard laparoscopic instruments. *Main Outcome Measure(s)*. Conversion to standard laparoscopic technique or laparotomy, estimated blood loss, operative time , extent of scarring, occurrence of intra- and perioperative surgical complications, technical adequacy, and clinical outcome. *Result(s)*. No conversion to standard laparoscopic technique or laparotomy, and no intraoperative or postoperative complications were observed. Total operative time was 99 minutes. The patient was discharged home on postoperative day one. *Conclusion(s)*. Laparoendoscopic single-site bilateral salpingo-oophorectomy of a large ovarian tumour is feasible with standard laparoscopic instruments. It is safe and effective, with good results in terms of excellent cosmesis and minimal postoperative pain.

## 1. Introduction

Laparoscopy has evolved rapidly over the past decade. We are witnessing a steady evolution towards progressively less invasive techniques. Although the adoption of robotic surgery has been hailed as a landmark in minimally invasive surgery, the huge initial capital outlay and the high maintenance costs are major obstacles. Recently, there is a renewed interest in single port gynaecological surgery, which was first reported by Wheeless in 1969, on the first single-incision tubal ligation [1]. However, laparoendoscopic single-site surgery (LESS) techniques did not take off initially due to limitations in the capabilities of laparoscopic equipment and imaging. 

Laparoendoscopic single-site surgery (LESS) techniques may be considered as a form of natural orifice transluminal endoscopic surgery (NOTES), via the umbilicus, which has recently emerged as a feasible form of minimally invasive procedure [2–4]. In fact, LESS techniques show comparable or better improvements in cosmesis and resulted in less postoperative pain than NOTES [5]. Currently, the LESS approach has been used mainly in the arenas of urologic and gastroenteric procedures such as nephrectomy [6], appendectomy [7], cholecystectomy [8], and hemicolectomy [9]. Reports on the use of LESS techniques in gynaecological surgeries are sparse [4]. Instrumentation to perform complex maneuvers intracorporeally are few, and several reports of single port surgery are at best considered as hybrid reports, in which the target organ was exteriorized through the umbilicus and extracorporeal open surgery performed [10–13].

The fundamental idea of single port surgery is to have all of the laparoscopic working ports enter the abdominal wall through the same incision [14, 15], further enhancing the cosmetic benefits of minimally invasive surgery while reducing the potential morbidity associated with multiple trocar incisions found in standard laparoscopic surgery [4, 6, 16, 17]. Naturally, LESS is not without its critics, common difficulties as evaluated in the initial case reports include partially compromised view arising from inline viewing, instrument crowding causing loss of “triangulation”, limited working space with hand collisions and crossing [10, 15].

In this paper, we describe our clinical experience and techniques for LESS in this first case of a bilateral salpingo-oophorectomy for a 10 cm ovarian fibroma, as well as outline our efforts in tackling the above-mentioned constraints imposed by single port surgery, and in doing so, hope to contribute to this exciting new area of laparoscopic surgery.

To our knowledge, this is the first case report in the region about LESS techniques being applied in this clinical scenario.

## 2. Case Summary

A 64-year-old Chinese lady first presented to our hospital with features of obstructive jaundice secondary to her underlying condition of choledocholithiasis, previously undiagnosed. Computed tomographic (CT) scan revealed a single adnexal mass of possibly ovarian origin. A gynaecological consult was sought and a detailed pelvic ultrasound was performed which showed a well-defined 10 cm solid mass posterior to and separate from the uterus, with low resistance vascularity on dopplers. The mass was highly mobile on clinical examination. 

The patient subsequently underwent an emergency laparoscopic cholecystectomy in view of her worsening jaundice and clinical status. A surveillance of the pelvis showed a large 10 cm left ovarian mass, well circumscribed and mobile, with features of an ovarian fibroma. The rest of her pelvic organs were grossly normal. As the duration of the emergency cholecystectomy was long due to surgical difficulties, the decision was made to remove the ovarian mass in a separate operation to avoid prolonging anaesthetic exposure. 

The operation was scheduled three months after her cholecystectomy. She remained asymptomatic, and the mass was constant in size. Open Hasson entry was performed and a 2 cm umbilical incision was made and concealed completely within the umbilicus to gain initial entry into the peritoneal cavity. The incision was then extended by another 0.5 cm via stretching of the skin. No other extraumbilical skin incisions were used. The entire umbilical scar measured 2.5 cm, which was just large enough to accommodate the single port.

**Figure 1:**
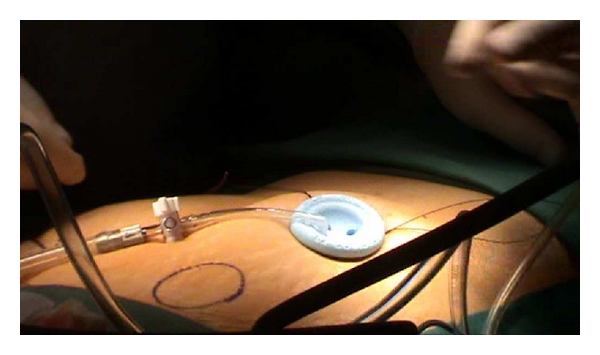
Creation of pneumoperitoneum.

**Figure 2:**
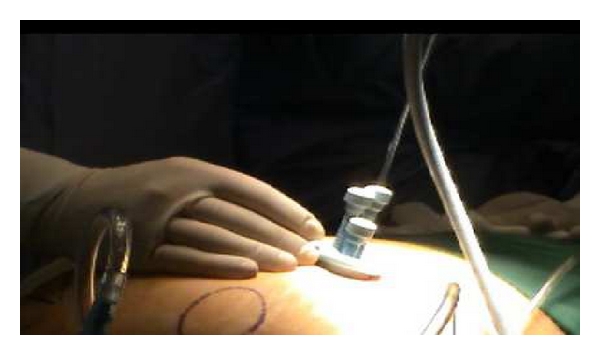
Mutliple port access.

**Figure 3:**
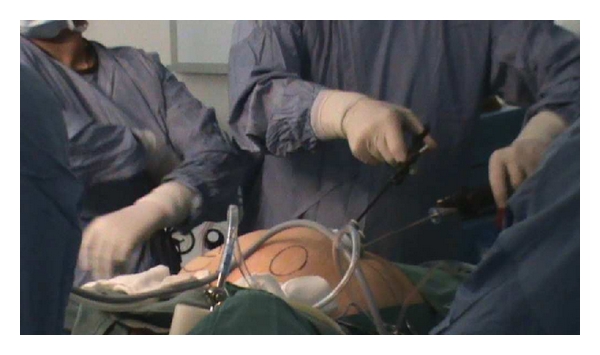
Instrumentation.

Next, the single port (Covidien) with three access inlets was introduced, and carbon dioxide pneumoperitoneum was created (Figure 1). A 5 mm rigid video laparoscope was deployed via one of the access port inlets (Figure 2), and other working instruments were introduced via the remaining two inlets (Figure 3). During the procedure, the patient was placed in Trendelenburg position. The uterus was manipulated with a Hegar dilator and the working instruments used were standard laparoscopic instruments.

Intra-abdominally, recreation of triangulation was done, which would be further elaborated in Section 3. Bilateral salpingo-oophorectomy was performed. The excised ovarian fibroma was placed into the Endocatch brought close to the umbilical port and morcellated to smaller segments before removal. Pneumoperitoneum was then deflated and ports were removed. Finally, the rectus sheath and skin were closed with vicryl. 

It was uncomplicated intraoperatively and there were no addition of ancillary ports nor conversion to laparotomy. The ovarian fibroma was removed completely with no residual tumour noted before closure. Cumulative blood loss was minimal. Operating time from incision to closure took 99 minutes. The entire procedure involved a three-man team inclusive of two surgeons and one assistant for uterine manipulation.

**Figure 4:**
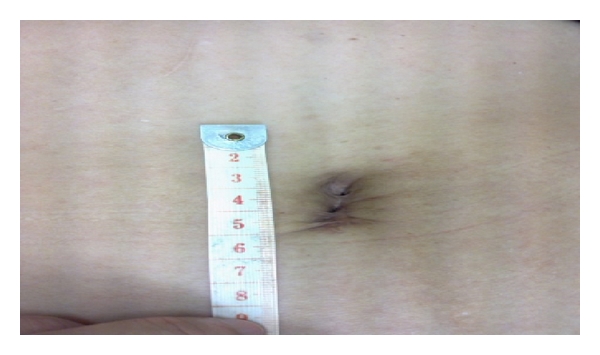
“Scarless” incision site (1 year postop).

Postsurgical recovery was uneventful and the patient was discharged well from inpatient observation on postoperative day one. There were no immediate surgical complications reported. Using the visual analogue scale, the patient reported a pain score of 1-2 immediately postsurgery. She required only oral analgesia for four days and was able to return to her full range of daily activities one week after the operation. Pain score never exceeded 2 during the postoperative period. The single surgical scar was well hidden in the umbilicus and patient reported high satisfaction level with postoperative cosmesis (Figure 4). There have been no other complications in the year after surgery.

## 3. Discussion

As with other surgeries conducted via single port access, we encountered similar technical challenges and constraints. The 10 cm size of the ovarian fibroma further contributed to the complexity of this case.

One of the biggest difficulties in single port surgery arises from the loss of triangulation. Wide spacing of trocars is a tenet of multitrocar standard laparoscopy. Parallel placement of instruments during single port surgeries makes triangulation difficult [10]. For this surgery, triangulation was achieved via several measures. Firstly, the SILS (Covidien) port is a blue flexible soft-foam port, with individual access channels for three cannulae (three 5-mm cannulas or two 5-mm and one 12-mm cannula). This design allows for greater maneuverability of the standard laparoscopic instruments to recreate triangulation intra-abdominally after entry through the umbilicus. Secondly, for pelvic surgery as in this case, uterine manipulation played a big role in facilitating operating positions for triangulation to be possible and also aided in providing traction. We made use of a single flexible/curved laparoscopic grasper to overcome parallel placement and recreate triangulation. Flexible and/or articulating instruments, which allow for intracorporeal triangulation, have been proposed as solutions to this problem [16]. However, bulk and technical challenge remain major obstacles in using articulating instruments at this stage of development [17]. 

Instrument crowding arises from a limitation in working space, as multiple instruments compete for the same space at the fulcrum of the entry port. This can result in hand collisions externally and difficulty with instrument tip manipulation internally [10, 15]. We attempted to maximize working space by holding the scope at a fixed distance away from the operating field. At this distance, we were able to achieve a fine balance between preventing the scope from interfering with the other operating instruments and yet not compromise on the field of vision. The problem is further aggravated by the surgeon's need to change the instruments multiple times during the surgery such as alternating the grasper with the bipolar. Perhaps the use of a single grasping diathermy would be useful in such circumstances, for example, Ligasure, PK knife, and so forth. Other multifunctional devices capable of grasping, dissecting, coagulating, and cutting can also overcome the limitations imposed by the reduced number of ports [16]. In the multi-institutional evaluation of LESS in gynaecology by Fader et al., multifunctional instruments (including the 5 mm Ligasure Advance (Covidien) or the Harmonic scalpel (Ethicon Endosurgery)) were utilized in all cases [4]. Further attempts have been made to perform LESS surgery via the da Vinci surgical system robotic platform [18–20]. Instruments with handles that can be articulated away from the port [15], or with varying lengths and streamlined profiles can also help avoid external hand collisions [10, 21, 22]. The limitation of lower excursion degrees among instruments in the abdominal cavity due to the loss of triangulation and instrument crowding was further hampered by the large size of the ovarian tumour. We worked around the constraints by shifting the traction maneuver from an orthogonal axis to a parallel one. 

Partially compromised view arising from inline viewing, associated with single port surgery [10, 15], was observed during the operation. Depth perception was lost as the camera lined up with the shaft of the working instrument [10]. Recent improvements of technologies such as flexible tip scopes (Olympus Endoeye) can minimize this restriction and emulate the stereoscopic vision offered by standard laparoscopic techniques [15]. This is achieved by a lower profile camera system such as the Olympus Endoeye, in which the video laparoscope is integrated with a coaxial light cable in line with the shaft of the telescope [17]. By creating an alternate camera angle with these flexible scopes, the camera is moved away from the shaft and other active surgical instruments. Angled telescopes also allow surgeons to experiment with placement of the camera so that it is placed in a position lateral to the working ports instead of the conventional umbilical position [15]. However, there are also varying opinions on the usefulness of flexible endoscopes in single port surgery due to its wavering when crossing the instruments [23]. In our surgery, a standard 5 mm rigid laparoscope was used, and it was effective in allowing us to perform the required procedure safely. 

The large size of the specimen also meant that intact removal from the single port access was very difficult. Morcellation using a Karl Storz Morcellator allowed us to reduce the fibroma into smaller sizes to enable easier removal. The excised specimen was first placed in a bag (Endocatch) introduced via the single port, brought close to the umbilical port, and morcellated into pieces. This allowed for faster removal of the pathological lesion, with reduction in total operative time. It was important to confine the morcellation process within the Endocatch, in view of the unknown histology of the ovarian mass, to avoid any possible peritoneal seeding if the mass indeed turned out malignant. Other means such as colpotectomy were technically feasible in removal of such a large ovarian mass, but were not adopted as we did not want to breach the peritoneal cavity in view of the potential malignancy of the tumour. 

Advantages associated with the usage of single port are largely derived from its excellent cosmesis result and improved quality of life postoperatively. With a hidden umbilical scar and no trocar incisions, excellent cosmetic result is achieved. Improved quality of life is similarly related to the elimination of multiple trocar sites, reducing morbidity related to visceral and vascular injury during trocar insertion, postoperative wound infection, and in the long-term, hernia formation [14]. The reduction of postoperative pain and analgesia usage has yet to be demonstrated for LESS surgery, due to a lack of comparative studies between single port and conventional laparoscopic surgeries. Evidently, the avoidance of multiple rectus muscle splitting incisions does result in faster recovery times and improved pain scores for patients. Careful selection of cases can prevent conversion to laparotomy, for example, low risk of malignancy, a nonobese patient with no history of more than two previous surgeries [4]. Extreme caution was also adopted during the assessment of the malignancy potential of the ovarian neoplasm during preoperative evaluation. Clinical examination, tumour marker panel, and detailed ultrasonographic investigations were performed for this patient. In this particular case, the surgeons also had the added benefit of performing a survey of the pelvic cavity and the tumour itself during the first cholecystectomy, which gave the team greater confidence to manage the neoplasm as a benign one despite the 10 cm size.

With regards to the surgical outcome, our operative time compares favourably to the series of 12 cases of embryonic natural orifice transumbilical endoscopic surgery (E-NOTES) for adnexal tumours performed by the Korean gynaecologic oncologists. The median operating time for the case series was 73 minutes, (range 25 to 110 minutes) and median blood loss was 10 ml (range 5 to 100 ml) [24], compared to 99 minutes for our procedure that involved removing a large 10 cm ovarian tumour and blood loss that was minimal in volume. No other complications were noted in the review one year postsurgery.

## 4. Conclusions

Laparoendoscopic single-site (LESS) salpingo-oophorectomy of a large ovarian tumor is feasible with standard laparoscopic instruments. We encountered similar difficulties and challenges during the operation, and hope to share our experience in tackling these problems. Some solutions that we proposed, such as recreation of triangulation and morcellation of tumour before removal, can be easily applied with the advancement of laproscopic technology. It is safe and effective, with good results in terms of excellent cosmesis and minimal postoperative pain. With more cases attempted in the future, the cost-effectiveness between the two methods may be further explored. As with any case of ovarian neoplasm, great caution should be exercised in evaluating the risk of malignancy before adopting LESS techniques.

It is believed that the role for single port laparopscopic surgery remains limited by the technical challenges originating from the breakdown in triangulation and instrument crowding [17]. Using this case as an example, we hope to illustrate possible measures to overcome this critical step and enable this surgical technique to play a bigger role in minimally invasive gynaecological surgery.

## References

[1] Wheeless CR (1969). A rapid, inexpensive, and effective method of surgical sterilization by laparoscopy. *The Journal of Reproductive Medicine*.

[2] Bessler M, Stevens PD, Milone L, Parikh M, Fowler D (2007). Transvaginal laparoscopically assisted endoscopic cholecystectomy: a hybrid approach to natural orifice surgery. *Gastrointestinal Endoscopy*.

[3] Rao MM, Rao RKM (2004). Two-port and single port loparoscopic appendicectomy. *Journal of the Indian Medical Association*.

[4] Fader AN, Rojas-Espaillat L, Ibeanu O, Grumbine FC, Escobar PF (2010). Laparoendoscopic single-site surgery (LESS) in gynecology: a multi-institutional evaluation. *American Journal of Obstetrics and Gynecology*.

[5] Kaouk JH, Haber GP, Goel RK (2008). Single-port laparoscopic surgery in urology: initial experience. *Urology*.

[6] Desai MM, Rao PP, Aron M (2008). Scarless single port transumbilical nephrectomy and pyeloplasty: first clinical report. *BJU International*.

[7] Ateş O, Hakgüder G, Olguner M, Akgür FM (2007). Single-port laparoscopic appendectomy conducted intracorporeally with the aid of a transabdominal sling suture. *Journal of Pediatric Surgery*.

[8] Gumbs AA, Milone L, Sinha P, Bessler M (2009). Totally transumbilical laparoscopic cholecystectomy. *Journal of Gastrointestinal Surgery*.

[9] Bucher P, Pugin F, Morel P (2008). Single port access laparoscopic right hemicolectomy. *International Journal of Colorectal Disease*.

[10] Canes D, Desai MM, Aron M (2008). Transumbilical single-port surgery: evolution and current status. *European Urology*.

[11] D’Alessio A, Piro E, Tadini B, Beretta F (2002). One-trocar transumbilical laparoscopic-assisted appendectomy in children: our experience. *European Journal of Pediatric Surgery*.

[12] Kosumi T, Kubota A, Usui N, Yamauchi K, Yamasaki M, Oyanagi H (2001). Laparoscopic ovarian cystectomy using a single umbilical puncture method. *Surgical Laparoscopy, Endoscopy and Percutaneous Techniques*.

[13] Cobellis G, Cruccetti A, Mastroianni L, Amici G, Martino A (2007). One-trocar transumbilical laparoscopic-assisted management of Meckel’s diverticulum in children. *Journal of Laparoendoscopic and Advanced Surgical Techniques*.

[14] Jung YW, Kim SW, Kim YT (2009). Recent advances of robotic surgery and single port laparoscopy in gynecologic oncology. *Journal of Gynecologic Oncology*.

[15] Romanelli JR, Earle DB (2009). Single-port laparoscopic surgery: an overview. *Surgical Endoscopy and Other Interventional Techniques*.

[16] Mereu L, Angioni S, Melis GB, Mencaglia L (2010). Single access laparoscopy for adnexal pathologies using a novel reusable port and curved instruments. *International Journal of Gynecology and Obstetrics*.

[17] Fader AN, Cohen S, Escobar PF, Gunderson C (2010). Laparoendoscopic single-site surgery in gynecology. *Current Opinion in Obstetrics and Gynecology*.

[18] Escobar PF, Fader AN, Paraiso MF, Kaouk JH, Falcone T (2009). Robotic-assisted laparoendoscopic single-site surgery in gynecology: initial report and technique. *Journal of Minimally Invasive Gynecology*.

[19] Joseph RA, Salas NA, Johnson C (2010). Chopstick surgery: a novel technique enables use of the da Vinci Robot to perform single-incision laparoscopic surgery. *Surgical Endoscopy and Other Interventional Techniques*.

[20] Joseph RA, Goh AC, Cuevas SP (2010). “Chopstick” surgery: a novel technique improves surgeon performance and eliminates arm collision in robotic single-incision laparoscopic surgery. *Surgical Endoscopy*.

[21] Escobar PF, Starks DC, Fader AN, Barber M, Rojas-Espalliat L (2010). Single-port risk-reducing salpingo-oophorectomy with and without hysterectomy: surgical outcomes and learning curve analysis. *Gynecologic Oncology*.

[22] Fagotti A, Fanfani F, Marocco F, Rossitto C, Gallotta V, Scambia G (2009). Laparoendoscopic single-site surgery (LESS) for ovarian cyst enucleation: report of first 3 cases. *Fertility and Sterility*.

[23] Fagotti A, Fanfani F, Rossitto C (2010). Laparoendoscopic single-site surgery for the treatment of benign adnexal disease: a prospective trial. *Diagnostic and Therapeutic Endoscopy*.

[24] Lim MC, Kim TJ, Kang S, Bae DS, Park SY, Seo SS (2009). Embryonic natural orifice transumbilical endoscopic surgery (E-NOTES) for adnexal tumors. *Surgical Endoscopy*.

